# Morphological and Molecular Characterization of KRAS G12C-Mutated Lung Adenocarcinomas

**DOI:** 10.3390/cancers14041030

**Published:** 2022-02-17

**Authors:** Radu Pirlog, Nicolas Piton, Aude Lamy, Florian Guisier, Ioana Berindan-Neagoe, Jean-Christophe Sabourin, Florent Marguet

**Affiliations:** 1 Pathology Department and INSERM U1245, Rouen University Hospital, Normandy University, 76000 Rouen, France; pirlog.radu@umfcluj.ro (R.P.); nicolas.piton@chu-rouen.fr (N.P.); aude.lamy@chu-rouen.fr (A.L.); florent.marguet@chu-rouen.fr (F.M.); 2 Research Center for Functional Genomics Biomedicine and Translational Medicine, “Iuliu Hatieganu” University of Medicine and Pharmacy, 400012 Cluj-Napoca, Romania; ioana.neagoe@umfcluj.ro; 3 Department of Pulmonology, Thoracic Oncology and Respiratory Intensive Care, Rouen University Hospital, 76000 Rouen, France; florian.guisier@chu-rouen.fr

**Keywords:** lung adenocarcinoma, KRAS, KRAS G12C, STK11, targeted therapy

## Abstract

**Simple Summary:**

Lung adenocarcinoma is currently the main histological subtype of lung cancer, accounting for more than 60% of diagnosed cases. The most frequent genomic alteration in these tumors is the mutation of the *Kirsten rat sarcoma viral oncogene homolog (KRAS)* gene, which until recently was not accessible to targeted therapy. New phase I, II, and III clinical trials using targeted inhibitors for the specific glycine-to-cysteine mutation at codon 12 (*KRAS c.34G>T/*KRAS G12C) of the *KRAS* gene showed promising results in approximately 30% of lung adenocarcinomas harboring a KRAS G12C mutation. In our study, we analyzed the genomic landscape of these tumors using next-generation sequencing technology and characterized new molecular subtypes that could be more susceptible to the new class of KRAS G12C inhibitors.

**Abstract:**

Lung adenocarcinoma (LUAD) is the major subtype of non-small cell lung cancer, accounting for approximately 60% of cases. Molecular analysis of LUADs showed that the *KRAS* gene is mutated in up to 30% of cases; such cases were previously considered “undruggable”. The KRAS G12C mutation has become a hot topic of research after initial, promising, phase I and II trials with targeted inhibitors. We analyzed the morphological and genomic landscape of 202 KRAS G12C mutated LUADs using next-generation sequencing, and identified a specific subtype of patients that could show an improved response to KRAS G12C inhibitors. The main histological subtype was acinar in 29.7% of cases. Tumor-infiltrating lymphocytes (TILs) were highly or moderately abundant in more than 60% of cases. The immunohistochemical profile showed TTF1 positivity in 78.7% of cases and PD-L1 positivity in 44.1% of cases. The molecular profile showed an association between *KRAS G12C* and *STK11* mutations in 25.2% of cases. This subgroup was associated with a statistically significant lower TTF1 (*p* = 0.0092) and PD-L1 (*p* < 0.0001) positivity. This type of combined morphological and molecular analysis can improve our understanding of tumor biology, and help us to identify specific patient subgroups that can achieve the best treatment response.

## 1. Introduction

Lung cancer is the second most common cancer and is responsible for more than 2.2 million cases and almost 1.8 million deaths per year worldwide [1]. Non-small cell lung cancers (NSCLC) are responsible for more than 85% of cases. The two main histological subtypes of NSCLC are squamous cell carcinoma and adenocarcinoma [2]. Molecular characterization of NSCLC has led to the identification of specific molecular alterations that can be successfully targeted by novel therapies [3]. Since the approval of targeted therapies against *EGFR* mutated lung adenocarcinomas (LUADs) by the US Food and Drug Administration (FDA), a dramatic increase in survival has been seen for these patients [4]. The most common activating EGFR mutations are the point mutation L858R and deletions in exon 19 [5]. These mutations, together with rearrangements in *ALK* and *ROS* genes, can be successfully targeted by tyrosine kinase inhibitors [6]. Additional functional mutations are found in *RET*, *MET,* and *BRAF* genes, which increase the number of actionable mutations for LUAD patients. These targeted therapies have led to major advances in LUAD therapy. However, targetable driver mutations are present in less than 50% of patients, and although patients showed initial response to these therapies, they frequently develop resistance. Therefore, new therapies and new actionable mutations are under investigation [7].

In the molecular landscape of LUADs, a mutation in the *Kirsten rat sarcoma viral oncogene homolog (KRAS)* gene is present in 30% of cases, making it the most frequent oncogenic driver mutation in this cancer type [8]. *KRAS*-mutated LUADs are a subgroup of cancers that are heterogeneous in their molecular background and clinical response to therapy [9]. Mutations in this oncogene are generally associated with a poor response to standard therapy. Numerous attempts to develop targeted molecules against *KRAS*-mutated cancers have been unsuccessful due to KRAS protein picomolar affinity for the abundance of cellular guanosine triphosphate (GTP) [10]. Among various mutations of this gene, the glycine-to-cysteine mutation at codon 12 (*KRAS c.34G>T/*KRAS G12C) is responsible for about 13% of *KRAS* mutations in LUADs, and is considered to be the most promising for the development of specific targeted molecules, as it facilitates binding to the cysteine amino acid of KRAS mutant protein [10,11]. The KRAS G12C mutation has become a major subject of interest after a new, small molecule (Sotorasib), which selectively and irreversibly targets the mutated oncoprotein, was successful in a phase I clinical trial [12]. Moreover, in a single-center phase II clinical trial on lung cancers harboring the KRAS G12C mutation, a complete response was seen in 3.2% of patients, and a partial response in 33.9% of patients, with a median duration of response of 11.1 months [13]. Adagrasib, a novel molecule acting on the same mechanism showed an overall response ratio of 45%, and a disease control rate of 96%, in the phase 1/2 KRYSTAL study (NCT03785249) [14]. Following publication of the clinical trial results, the FDA granted Breakthrough Therapy Designation in June 2021 [15]. As of February 2022, clinicaltrials.gov lists three clinical phase III trials (NCT05132075, NCT04685135, NCT04303780), evaluating KRAS G12C inhibitors in advanced and metastatic NSCLC harboring the specific KRAS G12C mutations that were previously treated with platinum-based chemotherapy and/or immune checkpoint inhibitor therapy.

In this study, we analyzed the clinical, histological, immunohistochemical, and molecular profiles of 202 KRAS G12C-mutated LUADs to advance current knowledge of this subset of lung cancers. This comprehensive characterization could improve our understanding of new molecular subtypes that can benefit from the improved response of currently available molecular targeted therapies.

## 2. Materials and Methods

### 2.1. Patient Samples

Pathology specimens from 202 patients whose surgical samples or lung biopsies were processed for molecular diagnosis between 2016 and 2020 in the Department of Pathology at Rouen University Hospital, France, were included in our study. All 202 patients were diagnosed with KRAS G12C mutated LUAD using DNA next-generation sequencing (NGS). Demographic data, such as sex and age at diagnosis, and clinical data regarding smoking status were collected, where available, from the hospital’s electronic health records.

### 2.2. Morphological Characterization

Tissue samples were collected during bronchial/lung biopsy or surgical intervention, then formalin-fixed and paraffin-embedded (FFPE). Each sample was individually assessed by two experienced pathologists for morphological parameters, including histology, percentage of tumor cells, nuclear atypia, necrosis, and tumor-infiltrating lymphocytes (TILs). Percentage of tumor cells was scored on a 5-level scale according to the institutional internal protocol for molecular pathology: <5%, 5–15%, 15–25%, 25–50%, and >50%. Nuclear atypia and tumor necrosis were scored on a dichotomous scale as present or absent. TIL abundance was scored on a 4-level scale: high—3, moderate—2, low—1, rare/absent—0.

### 2.3. Immunohistochemistry

Routine diagnostic immunohistochemistry (IHC) for LUAD was performed on a Ventana Benchmark© GX (Roche^®^, Rotkreuz, Switzerland) for thyroid transcription factor 1 (TTF1) (Clone SP141, Roche^®^—prediluted), p40 (Clone BC 28, Roche^®^—prediluted), CK7 (Clone SP52, Roche^®^—prediluted), and programmed death-ligand 1 (PD-L1) (Clone QR1, Diagomics^®^, Blagnac, France—dilution 1/100). Immunohistochemistry staining for PD-L1 and TTF1 was scored following the WHO classification of lung tumors (5th edition); a case is considered positive if at least 1% of tumor cells are positive [2]. Tumors were categorized using a dichotomous scale as either positive or negative after examining the whole slide. Each IHC slide was individually assessed by two experienced pathologists. Where discordance was found, the cases were assessed by a panel and a consensus was reached.

### 2.4. Next-Generation Sequencing

Molecular analysis was performed on FFPE samples with a minimum tumor cellularity of 5%. Tumor DNA was extracted using the Maxwell^®^ 16 FFPE Plus LEV DNA Purification kit on a Maxwell^®^ 16 Instrument (Promega^®^, Madison, WI, USA). NGS analysis was performed on a MiSeq sequencer (Illumina©, San Diego, CA, USA). The gene library was prepared with the Tumor Hotspot MASTR Plus kit (Multiplicom^®^, Niel, Belgium). The regions analyzed were: *AKT1 (exon 3), ALK (exons 20–29), BRAF (exons 11, 15), CDKN2A (exons 1–3), CTNNB1 (exon 3), DDR2 (exons 3–18), EGFR (exons 18–21), ERBB2 (exons 19–21), ERBB4 (exons 10–12), FGFR2 (exons 7, 10, 12), FGFR3 (exons 7, 9, 14, 15), H3F3A (exon 2), HIST1H3B (exon 1), HRAS (exons 2–4), IDH1 (exon 4), IDH2 (exon 4), KIT (exons 8, 9, 10, 11, 13, 14, 17, 18), KRAS (exons 2–4), MAP2K1 (exons 2, 3), MET (exons 2, 10, 14–20), NRAS (exons 2–4), PDGFRA (exons 12, 14, 18), PIK3CA (exons 2, 3, 10, 11, 21), PIK3R1 (exons 11, 12, 13), PTEN (exons 1–9),* and *STK11 (exons 1–9).* Sequencing data were analyzed with BWA-GATK-07.6a-3.1.1 software, variant identification was performed with VarScan2, and data were recorded with Alamut HT^®^ software. The limit of detection was 2% of mutated reads for a minimum of 30 reads/mutation. NGS results were individually assessed by two experienced molecular biologists/pathologists. The allelic ratio was quantified using NGS software. The expected allelic ratio corresponds to half the percentage of tumor cells, for example, a sample containing 25–50% of tumor cells corresponds to an expected allelic ratio between 12.5–25%. A mismatch between tumor cellularity and KRAS G12C allelic ratio was considered as present when there was a difference of more than 10% between tumor cellularity evaluated by hematoxylin-eosin staining and allelic ratio based on NGS analysis.

### 2.5. Statistical Analysis

Statistical analysis was carried out using GraphPad Prism software, version 8.3.1 (GraphPad, San Diego, CA, USA). To compare differences between the KRAS G12C-STK11 co-mutated LUAD and KRAS G12C-nonSTK11 mutated LUAD subgroups the chi-squared test and Fisher’s exact test were used. *P*-values lower than 0.05 were considered statistically significant.

## 3. Results

### 3.1. Demographic Characteristics

The mean age in our patient group was 62.5 years (42–83 years). The sex distribution was 129 men (63.9%) and 73 women (34.1%). Smoking status was available for 44 out of 202 patients; 43 of these patients were smokers and one was a nonsmoker, suggesting that a large majority of our patients are smokers.

### 3.2. Morphology and Immunohistochemistry

**Figure 1:**
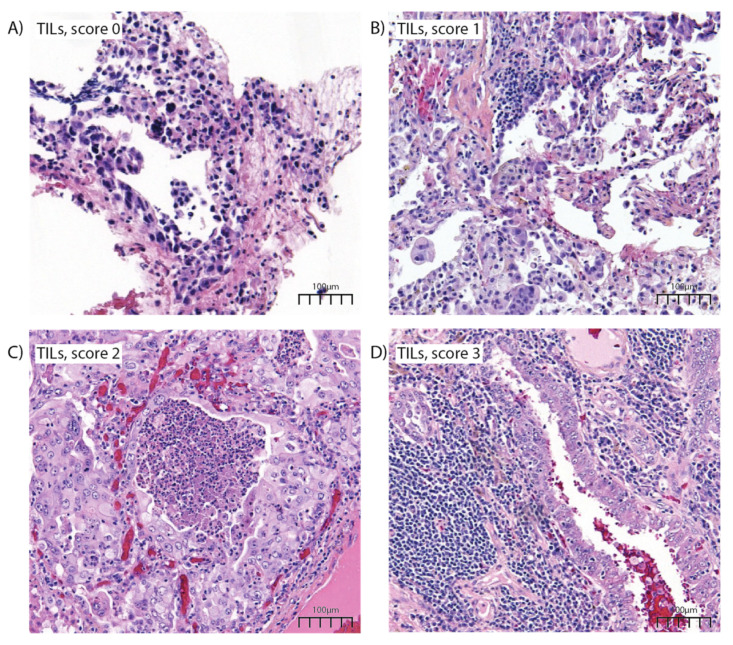
Hematoxylin and eosin staining of lung adenocarcinomas, 100× magnification. (**A**) LUAD showing rare TILs, score 0. (**B**) LUAD with a low abundance of TILs, and cancer cells with atypical nuclei. (**C**) LUAD with a moderate abundance of TILs, intratumor necrosis, and atypical nuclei. (**D**) LUAD with a high abundance of TILs, TILs are enclosing the tumor islands in an attempt to limit spreading of the tumor.

Morphological characterization of samples showed acinar histology in 29.7%, a high abundance of TILs in 37.6%, presence of intratumor necrosis in 32.6%, and presence of nuclear atypia (Figure 1) in 71.2% (Table 1).

**Figure 2:**
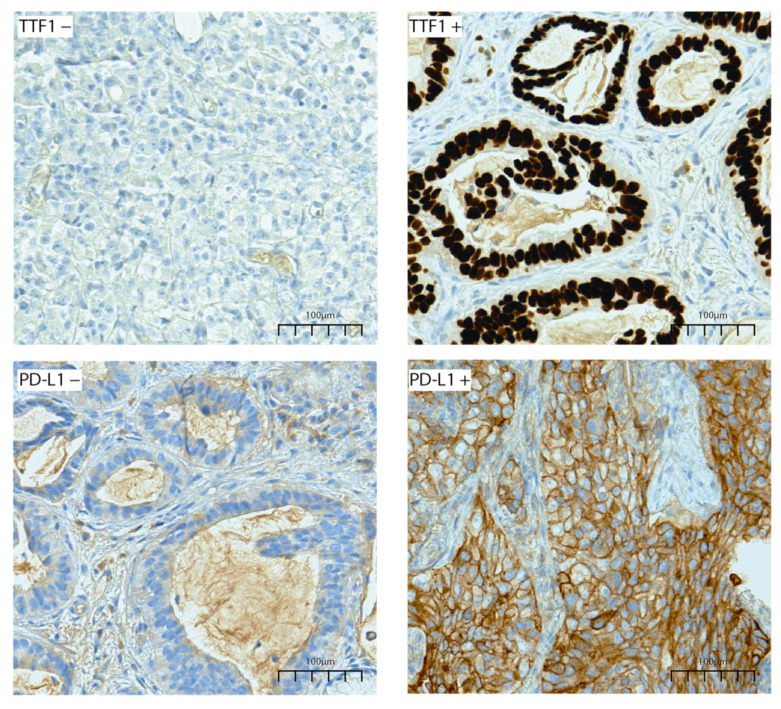
Immunohistochemistry staining for TTF1 and PD-L1 in lung adenocarcinoma samples, 200× magnification. The TTF1 marker is showing an intensively positive nuclear staining. The PD-L1 marker is highlighting a positive continuous membranous staining.

Immunohistochemistry staining showed TTF1 positivity in 78.7% of cases and PD-L1 positivity in 44.1% of samples (Table 1) (Figure 2).

### 3.3. Next-Generation Sequencing Analysis

NGS analysis identified the *KRAS c.34G>T/*KRAS G12C mutation in all analyzed samples. Among these, 101 cases were associated with one additional mutation in another gene, 24 cases were associated with two additional mutations, and 5 cases were associated with three or more additional mutations (Table 2). A mismatch between KRAS G12C mutation allelic ratio and tumor cellularity was found in 24.3% of the analyzed samples.

### 3.4. KRAS G12C and STK11 Co-Mutated LUADs

Following NGS analysis, a frequent association was observed between KRAS G12C and mutations in the *STK11* gene in 51 samples (25.2%). *STK11* was the only mutation in 40 of these samples, and was associated with one other mutated gene in 8 and two other mutated genes in 3 of them. Morphological characterization of this co-mutated subgroup showed acinar histology in 39.5%, a high abundance of TILs in 39.2%, presence of necrosis in 23.5%, and presence of atypia in 68.6% of samples. The immunohistochemistry characteristics of the co-mutated subgroup include TTF1 positivity in 64.7% and PD-L1 positivity in 19.6% of samples (Table 1). PD-L1 and TTF1 IHC expression in the KRAS G12C-STK11 co-mutated subgroup was significantly lower when compared with the KRAS G12C-nonSTK11 mutated samples (*p* < 0.0001 for PD-L1 and *p* = 0.0092 for TTF1).

## 4. Discussion

KRAS G12C is an activator mutation of the *KRAS* oncogene leading to increased levels of mutated KRAS oncoprotein and enhanced tumor viability [16]. Currently, in KRAS-mutated LUADs, the standard treatment includes immune-checkpoint inhibitors, either in monotherapy, or in combination with chemotherapy; but, in the absence of response, or in the presence of disease progression, the options for second-line treatment are limited [17,18]. The results from the phase II trial on NSCLC treated with sotorasib were promising, showing reversible toxic effects and disease control in 80% of patients, with a median progression-free survival of 6.8 months and a median overall survival of 12.5 months [13]. Similar results were shown with adagrasib in the KRYSTAL trial [14]. These two molecules are approved by the FDA for clinical use in KRAS G12C mutated locally advanced or metastatic NSCLC that have already received at least one line of systemic therapy [19]. The objective response rate for the two molecules in currently published studies is between 37.1% and 43% [13,14]; therefore, there is a need for a better stratification of the patients who could benefit from these new molecular targeted therapies.

Recently, several studies analyzed the possible mechanisms of resistance to these targeted therapies, indicating various mechanisms that could explain the limited response of these novel inhibitors. One such mechanism is the low tumoral addiction of the mutated KRAS protein, indicating that in the case of a KRAS blockade the main canonical growth pathways (MAPK/ERK, PI3K/AKT/mTORC1) will be activated through alternative pathways [20,21]. The KRAS G12C mutation can co-occur with other genetic alterations that are not targeted by the inhibitor molecule, which could continue to promote tumor growth and treatment resistance [22]. For example, CDKN2A mutations which were identified in 6.9% of samples were recently associated with increased resistance to immune checkpoint inhibitors. A study by Gutiontov et al. showed that NSCLC harboring a CDKN2A loss-of-function mutation had a twofold increase in immunotherapy resistance, irrespective of PD-L1 expression, and a poorer clinical outcome [23]. In addition, *DDR2* gene mutations were associated with 4.5% of our cases; DDR2-mutated LUADs showed sensitivity to dasatinib, supporting a possible role for combined chemotherapy in these patients [24]. In vitro studies on NSCLC KRAS G12C-mutated cell lines showed that the co-existence of *MET* amplification is responsible for resistance to sotorasib treatment in mutated cell lines. Interestingly, the chemosensitivity to sotorasib treatment is restored by the association with a *MET* inhibitor, crizotinib [25]. In our study, the KRAS G12C mutation was associated with a *MET* mutation in four cases, indicating that there might be a need for adaptive treatment regimens according to the specific mutational profile of the tumor. On the other hand, a chemotherapy-resistant subgroup is represented by the KRAS–ALK co-mutated tumors; we identified four cases in our cohort, which are associated with acquired resistance to crizotinib [26,27].

Another mechanism of KRAS G12C treatment resistance is due to intratumor heterogeneity, with the *KRAS* mutation co-existing with various mutations in different tumor clones; combinations of driver mutations in LUAD is generally associated with increased resistance to TKI, and targeted therapies should be considered during treatment [28,29].

An interesting result in our cohort is the absence of KRAS G12C-EGFR co-mutations. These two driver mutations have been described as being mutually exclusive by multiple studies analyzing large study samples [7,30,31]. The rare occurrence of KRAS G12C-EGFR co-mutations are represented by nonstandard rare mutations [7].

In our study, we detected co-existing mutations in 50% of the samples studied. Additionally, four cases in our cohort showed two different *KRAS* gene mutations, suggesting either the existence of two tumor clones, one of which cannot be targeted by KRAS inhibitors, or—in the case that both mutations occur in the same tumor cells—that the mutated KRAS protein structure might not be targeted with the same efficiency. Furthermore, we detected a mismatch between the tumor cellularity and the allelic ratio of the KRAS G12C mutation in 24.3% of cases, suggesting that, at the cellular level, the mutation is heterogeneously present among tumor cells. This intratumor heterogeneity could explain the relatively lower response of KRAS inhibitors, as they may target only a subtype of tumor cells. New studies focusing on intratumor heterogeneity should evaluate the correlation between the distribution across tumor cells of the KRAS G12C mutation and the response to its targeted inhibitors.

Additionally, data from the CodeBreaK100 Clinical Trial showed that co-occurring mutations in *TP53*, *KEAP1,* and *STK11* genes could interfere with the efficacy of KRAS G12C inhibitors [13]. *STK11* and *KEAP1* co-mutations in KRAS-mutated NSCLC are associated with a poor prognosis and resistance to anti-PD1 and anti-PD-L1 blockade [30,31,32]. Immune checkpoint inhibitors were shown to negatively impact the overall survival of patients harboring KRAS-STK11 co-mutations, highlighting the importance of NGS sequencing for better treatment stratification [33]. On the other hand, in the KRYSTAL 1 and CodeBreaK100 clinical trials, patients who harbored KRAS G12C and STK11 co-mutations showed a higher response rate for single agent adagrasib and sotorasib [13]. Despite the small sample size of the clinical trial, the results are noteworthy as they show promise for a group of patients previously considered to have a limited chance of treatment response. In our study, we were able to characterize a distinct subgroup of KRAS G12C-STK11 co-mutated tumors in 25% of our cases, which is similar to data reported by other groups in the literature [34]. The morphological characterization showed a higher prevalence of the acinar phenotype in this subgroup (39.3% versus 26.5%), similar abundance in TILs with only minor differences between the two groups, lower intratumor necrosis (23.5% vs. 35.8%), and similar presence of nuclear atypia. These KRAS G12C-STK11 co-mutated tumors showed particular IHC phenotypes with lower TTF1 (*p* = 0.0092) and lower PD-L1 expression (*p* < 0.0001) when compared with KRAS G12C-nonSTK11 mutated LUADs. The significantly lower PD-L1 immunohistochemistry positivity in the KRAS G12C-STK11 co-mutated subgroup is in accordance with the known resistance of these tumors to anti-PD-L1 treatment [30]. Studies with mice show that KRAS G12C inhibitors induce a pro-inflammatory phenotype in the tumor microenvironment of LUADs, which can be further exploited with targeted anti-PD-1 therapy [35,36]. Therefore, new therapeutic strategies are emerging for these tumors by combining the targeted KRAS G12C inhibitors with the anti-PD-1 antibody pembrolizumab (NCT03600883, NCT04613596, and KRYSTAL-7).

We present a retrospective analysis of 202 KRAS G12C mutated LUADs diagnosed in our Department of Pathology. Using a combined morphological and molecular approach we characterized the histological aspects of these tumors, including the tumor microenvironment landscape and the main molecular alterations associated with the KRAS G12C mutation. A potential limitation of our study is related to the lack of clinical data regarding the oncologic treatment of these patients and correlation of the molecular landscape with treatment and overall survival.

Future prospective studies and clinical trials should analyze the response to KRAS G12C inhibitors in distinct subgroups of patients harboring complex genomic landscapes to identify responders and non-responders according to their specific molecular signature. Current clinical trials are stratifying patients only according to primary tumor site and KRAS G12C mutation. This limited molecular classification of tumors might lead to false negative trials by including in the same trial patients with co-occurring mutations that can significantly influence the metabolism and resistance of the tumor to targeted molecules.

## 5. Conclusions

Our morphological, immunohistochemical, and molecular study presents a cohort of 202 cases of KRAS G12C-mutated LUADs. Morphological characterization has shown that these subgroups of tumors have an active tumor microenvironment with a moderate to high abundance of TILs in the majority of cases and a PD-L1 positivity in 44% of samples. Molecular analysis highlighted the complex co-existence of genomic alterations in various genes including *MET* and *STK11.* Additionally, a subgroup of tumors harboring both *KRAS G12C* and *STK11* mutations emerged during the molecular analysis with specific morphological and immunohistochemistry characteristics. This subgroup of patients, which is known for its limited response to anti-PD-L1 treatment, might benefit from the new KRAS G12C inhibitors.

An in-depth characterization of KRAS G12C-mutated LUADs is essential as we shift from the previous “undruggable” *KRAS* into the new era of targeted therapeutics. There is a need for a more in-depth genomic diagnosis of patients with complex tumor genomic backgrounds as this information can help clinicians better stratify patients for currently available therapies and identify the subgroups of patients that could benefit from combinatorial therapies or alternative approaches.

## Figures and Tables

**Table 1:** Morphological and immunohistochemical characteristics of KRAS G12C mutated LUADs. Data are presented individually for the KRAS G12C-STK11 co-mutated LUADs subgroup and the KRAS G12-nonSTK11 mutated LUADs subgroup.

Variable/Molecular Subtype	Total Cases	KRAS G12C-STK11 Co-Mutated LUADs	KRAS G12C-nonstk11 Mutated LUADs	*p*-Value
	(*n* = 202 Cases)	(*n* = 51 Cases)	(*n* = 151 Cases)	
**Histology**				0.3263
acinar	60 (29.7%)	20 (39.3%)	40 (26.5%)	
solid	36 (17.8)	6 (11.8%)	30 (19.9%)	
lepidic	15 (7.5%)	4 (7.8%)	11 (7.3%)	
mucinous	7 (3.4%)	2 (3.9%)	5 (3.3%)	
poorly differentiated	8 (3.9%)	0 (0%)	8 (5.3%)	
material of cytologic value	26 (12.9%)	8 (15.7%)	18 (11.9%)	
NOS *	50 (24.8%)	11 (21.5%)	39 (25.8%)	
**Tumor cellularity**				0.8789
>50%	40 (19.8%)	9 (17.6%)	31 (20.6%)	
25–50%	55 (27.2%)	14 (27.5%)	41 (27.2%)	
15–25%	83 (41.1%)	23 (45.1%)	60 (39.7%)	
5–15%	24 (11.9%)	5 (9.8%)	19 (12.5%)	
TILs				0.921
3+	76 (37.7%)	20 (39.2%)	56 (37.1%)	
2+	58 (28.7%)	14 (27.5%)	44 (29.1%)	
1+	37 (18.3%)	8 (15.7%)	29 (19.2%)	
0+	31 (15.3%)	9 (17.6%)	22 (14.6%)	
**Intratumor necrosis**				0.1222
Present	66 (32.3%)	12 (23.5%)	54 (35.8%)	
Absent	136 (67.7%)	39 (76.5%)	97 (64.2%)	
**Nuclear atypia**				0.7206
Present	144 (71.2%)	35 (68.6%)	109 (72.2%)	
Absent	58 (28.8%)	16 (31.4%)	42 (27.8%)	
**TTF1 IHC**				0.0092
Positive	159 (78.7%)	33 (64.7%)	126 (83.4%)	
Negative	43 (21.3%)	18 (35.3%)	25 (16.6%)	
**PD-L1 IHC**				<0.0001
Positive	89 (44.1%)	10 (19.6%)	79 (52.3%)	
Negative	113 (55.9%)	41 (80.4%)	72 (47.7%)	

* The term NOS (not otherwise specified) was used for biopsy samples in which it was difficult to assign a specific histologic pattern to the histology specimen.

**Table 2:** Additional mutated genes in KRAS G12C mutated LUADs.

Mutated Gene	Cases, *n* (%)	Mutated Gene	Cases, *n* (%)
*ALK*	4 (1.9%)	*IDH2*	1 (0.5%)
*BRAF*	1 (0.5%)	*KIT*	2 (1%)
*CDK2NA*	15 (7.5%)	KRAS *	4 (2%)
*CTNNB1*	7 (2.5%)	*MAPK*	2 (1%)
*DDR2*	9 (4.5%)	*MET*	4(2%)
*FGFR2*	1 (0.5%)	*NRAS*	2 (1%)
*FGFR3*	3 (1.5%)	*PIK3CA*	15 (7.4%)
*H3F3A*	2 (1%)	*PTEN*	5 (2.4%)
*HIST1H3B*	4 (2%)	*STK11*	51 (25.2%)

* for *KRAS* gene the 4 cases present another mutation in addition to KRAS G12C.

## Data Availability

The data underlying this article cannot be shared publicly to maintain the privacy of individuals that participated in the study. The data will be shared upon reasonable request to the corresponding author.
